# Effect of Different Matrix Metalloproteinase Inhibitors on the Shear Bond Strength of Composite Attached to Dentin: An In Vitro Study on Permanent Teeth

**DOI:** 10.7759/cureus.95394

**Published:** 2025-10-25

**Authors:** Poulami Chaudhuri, Chi Koy Wang, Somnath Pal, Abhinav K Singh, Ananjan Chatterjee, Karthikeyan Ramalingam, Abhishek Banerjee

**Affiliations:** 1 Department of Conservative Dentistry and Endodontics, Buddha Institute of Dental Sciences and Hospital, Patna, IND; 2 Department of Oral and Maxillofacial Pathology, Buddha Institute of Dental Sciences and Hospital, Patna, IND; 3 Department of Oral Pathology and Microbiology, Malla Reddy Institute of Dental Sciences, Malla Reddy Vishwavidyapeeth, Hyderabad, IND; 4 Department of Oral and Maxillofacial Pathology, Awadh Dental College and Hospital, Jamshedpur, IND

**Keywords:** chlorhexidine, composite resins, dentin-bonding agents, gluma desensitizer, matrix metalloproteinase, matrix metalloproteinase inhibitors, permanent tooth, proanthocyanidin, shear bond strength, utm

## Abstract

Aim: This study aimed to evaluate the effect of various matrix metalloproteinase (MMP) inhibitors on the shear bond strength (SBS) of composite resin to dentin in permanent teeth using an etch-and-rinse adhesive system and the pairwise comparative analysis between each MMP inhibitor group and the control group.

Materials and methods: Dentin blocks were prepared from 60 permanently extracted molars and were randomly distributed into four groups (each group, n = 15). The dentinal blocks were pretreated with 0.01M phosphate-buffered saline (PBS) (pH 7.2) in the control group (Group I), 2% chlorhexidine (CHX) (Group II), Gluma desensitizer (Kulzer, Germany) (Group III), and 6.5% proanthocyanidin (Group IV) before applying the etch and rinse adhesive system (Ivoclar, Switzerland). After the adhesive application, composite resin (Kulzer, Germany) was applied, and after seven days, the SBS values were determined with a Universal Testing Machine (Zwick/Roell Z020, ZwickRoell Group, Germany).

Results: There was a significant difference in the SBS values of Gluma desensitizer, 2% CHX, 6.5% proanthocyanidin, and the control group (P < 0.05). The Gluma desensitizer group showed the highest bond strength values (mean 12.98 MPa), which were significantly greater than those of all the other groups.

Conclusions: Under the experimental conditions of this study, pretreatment with MMP inhibitors was advantageous in improving the SBS of the composite attached to permanent teeth dentin, with Gluma being most effective.

## Introduction

Introduced in the mid-20th century, the concept of adhesive dentistry marked a significant breakthrough, initiating a paradigm shift from traditional metal-based restorations. This transition has revolutionized the field of restorative dentistry. Adhesion between the tooth substrate and resin-based materials plays a crucial role in the success of long-lasting restorations [1]. Researchers have observed that bonding to dentin presents greater challenges than bonding to enamel, and achieving long-lasting adhesion to dentin has imposed a considerable obstacle in the field of restorative dentistry [2]. It is seen that resin monomers infiltrate into the demineralized dentin to form a hybrid layer. Despite its intended function, it is frequently the most vulnerable zone of adhesion, thereby limiting bond durability [1,3].

In etch-and-rinse adhesives, the primer, containing hydrophilic monomers, infiltrates the exposed collagen network, and the bonding agent resin forms a thicker hybrid layer compared to the typically thinner, more superficial hybrid layer in self-etch adhesive systems [4,5]. Consequently, the etch-and-rinse system is known for offering the most reliable and stable performance in evaluating shear bond strength (SBS) at the resin-dentin interface and was selected for this study [6]. Researchers emphasize that SBS is a crucial parameter in evaluating the stability, durability, and overall integrity of the bond formed between the adhesive and the tooth structure [7].

Endogenous matrix metalloproteinases (MMPs) belong to a family of host-derived, calcium- and zinc-dependent enzymes trapped in the dentin matrix during tooth formation. They frequently break down the exposed and brittle collagen fibrils of dentin by hydrolysis of the extracellular matrix. MMPs are activated by the acidic environment of cavities on the tooth surfaces [8,9].

Therefore, long-term bonding and stability remain a concern, as they are threatened by the degradation of the hybrid layer, which in turn reduces bond strength, primarily due to MMP activation. To counter this, pretreating the bonding interface with MMP inhibitors like chlorhexidine (CHX) [6], ethylenediaminetetraacetic acid (EDTA) [10,11], tetracycline (TC) [12], Gluma desensitizer (Kulzer, Germany), containing 5% glutaraldehyde and 35% 2-hydroxyethyl methacrylate (HEMA) [13], and 6.5% proanthocyanidin [14] are crucial. CHX effectively inhibits MMP-2, 8, and 9, with 2% CHX being suggested as optimal [15]. Around 6.5% proanthocyanidin (grape seed extract) is notable in conservative dentistry as a cross-linking agent and for inhibiting the production and catalytic activity of several MMPs (MMP1, 2, 3, 7, 8, 9, and 13) [14]. Gluma is known for its antibacterial properties and for reducing postoperative sensitivity, also inhibiting MMP-2, 8, and 9 in human dentin [13].

Literature searches have limited data on the effects of MMP inhibitors like 2% CHX, 6.5% proanthocyanidin, and Gluma on the adhesive properties of permanent dentition. A comprehensive analysis that includes both intergroup and pairwise comparisons of their influence on SBS has not yet been conducted, necessitating further investigation. Therefore, this study aims to evaluate a few MMP inhibitors and their impact on the SBS of the composite.

## Materials and methods

Sample preparation

The study was conducted at the Buddha Institute of Dental Sciences and Hospital, Patna, India. Around 60 extracted sound human permanent molars free of discoloration, carious lesions, defects, and endodontic treatments that have been extracted for periodontal/prosthetic/orthodontic reasons were selected for this study after approval from the institutional ethical committee (approval number: 132/BIDSH, Date: July 15, 2023). G*Power software (Version 3.1.9.7, Heinrich-Heine-Universität Düsseldorf, Düsseldorf, Germany) was used to calculate the sample size. A sample size of 15 specimens per group (four groups; total n = 60) was chosen. This decision was based on previous literature. This is supported by the SBS literature, which widely accepts a sample size of n = 10-15 per group for in vitro adhesion studies [16,17]. For the key comparison of interest (Gluma desensitizer vs. proanthocyanidin), means and standard deviations (Gluma: 12.9847 ± 0.2371 MPa; proanthocyanidin: 9.3187 ± 0.3020 MPa) yielded an estimated effect size of Cohen’s d = 13.5. Using G*Power version 3.1.9.7 with a two-tailed test, α = 0.05, and 90% power, the minimum required sample size per group was calculated to be n = 15. This value was adopted to ensure 90% statistical power.

The materials used in the study included phosphate-buffered saline (PBS), composed of sodium chloride, disodium hydrogen phosphate, potassium chloride, and potassium dihydrogen phosphate (Batch No. 4399406). A 2% CHX digluconate solution in isopropyl alcohol (Batch No. 0624 2026-06) was also employed. Gluma desensitizer, containing 5% glutaraldehyde and 35% HEMA (Batch No. 10559), was used as a desensitizing agent. Additionally, a 6.5% proanthocyanidin solution was prepared by dissolving 6.5 g of grape seed extract in 100 ml of distilled water (Batch No. ILGS-144).

**Figure 1:**
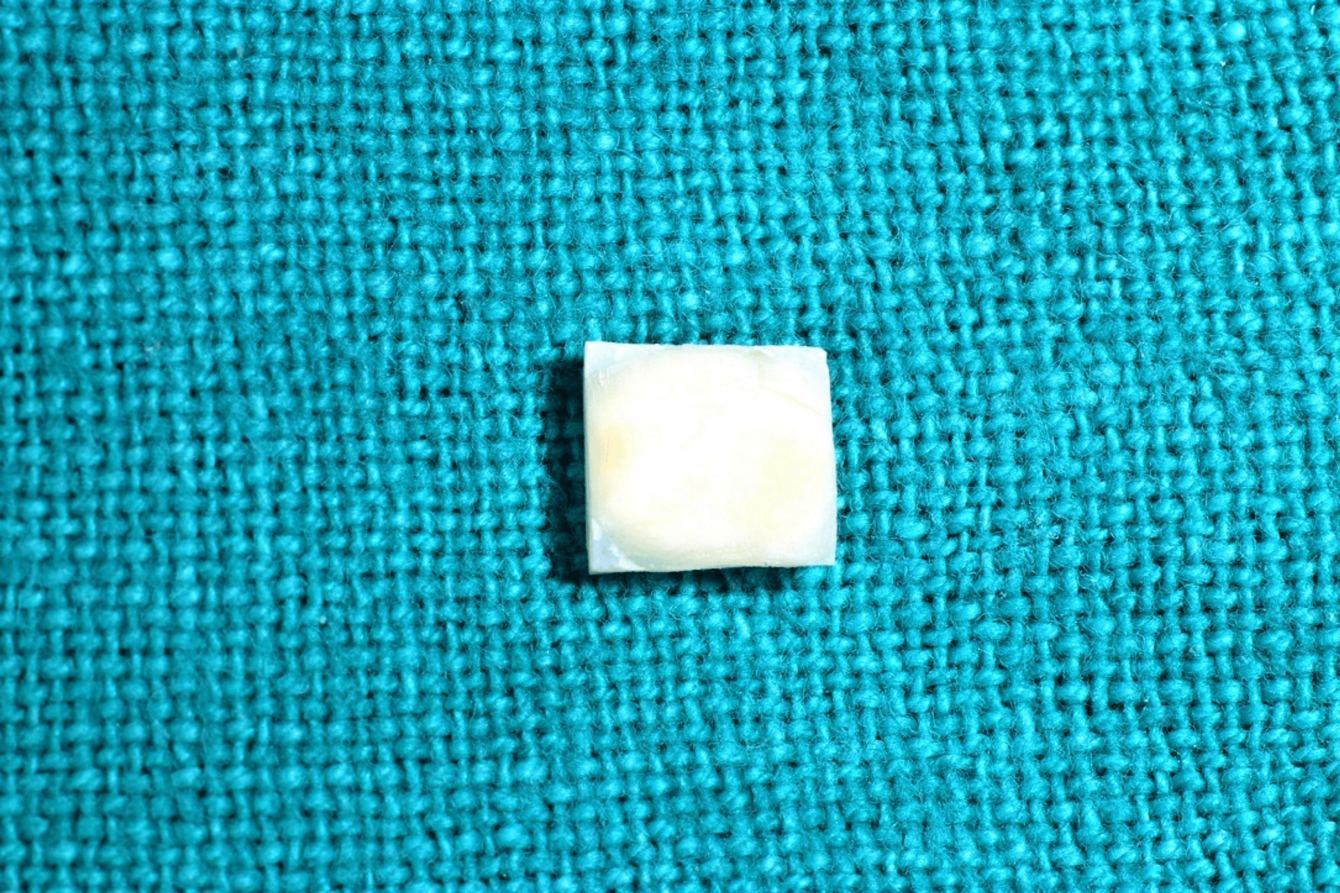
Dentin block created from each tooth

From each tooth, a dentin block measuring 6.0 mm × 6.0 mm × 2.0 mm was obtained (Figure 1).

**Figure 2:**
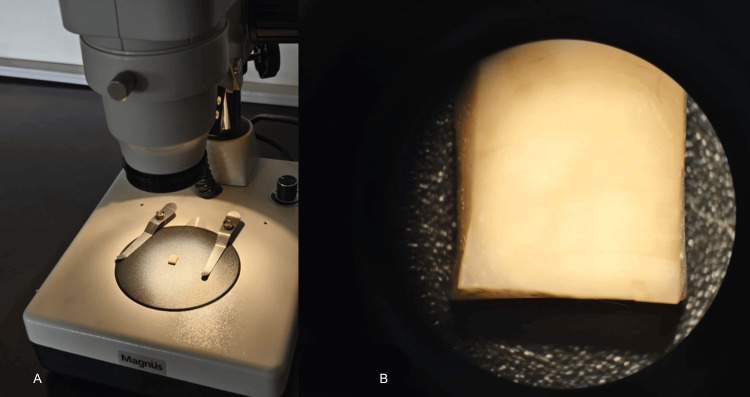
Dentin block viewed under a stereomicroscope for enamel residues A: the sample placed on the stereomicroscope; B: the photomicrograph at 25x magnification

A stereomicroscope at a magnification of 25X was utilized to view the block, and the lack of enamel remnant was verified (Figure 2).

Before specimen testing, the examiner underwent standardization and calibration. A set of 10 pilot specimens was randomly selected, and SBS was measured twice on each specimen under identical conditions, with a 48-hour interval between sessions to prevent recall bias. For intra-examiner reliability, repeat measurements were performed after complete specimen re-mounting and machine realignment. The dentin blocks were etched with 37% phosphoric acid (Eco-Etch, Ivoclar, Vivadent, Switzerland) for 30 seconds to create the acid conditioning procedure, followed by thorough washing in water. Dentin blocks (N = 60) were then randomly divided into four groups (n = 15).

Group I (control group) was pretreated with PBS 0.01M, having a pH of 7.2 (Sisco, Research Laboratories Pvt Ltd, India) for 60 seconds. Group II (study group) was pretreated with 2% CHX gluconate (Hexachlor, Safe Endo, India) for 60 secs. Group III (study group) was pretreated with Gluma desensitizer for 30 secs. Group IV (study group) was pretreated with 6.5% proanthocyanidin (Inlife, India) for 60 seconds after etching with 37% phosphoric acid. Based on previous studies, a 6.5% concentration of proanthocyanidin was used, as it has been shown to effectively inhibit dentin MMPs, enhance collagen stability, and improve SBS, while maintaining favorable handling characteristics for adhesive application.

**Figure 3:**
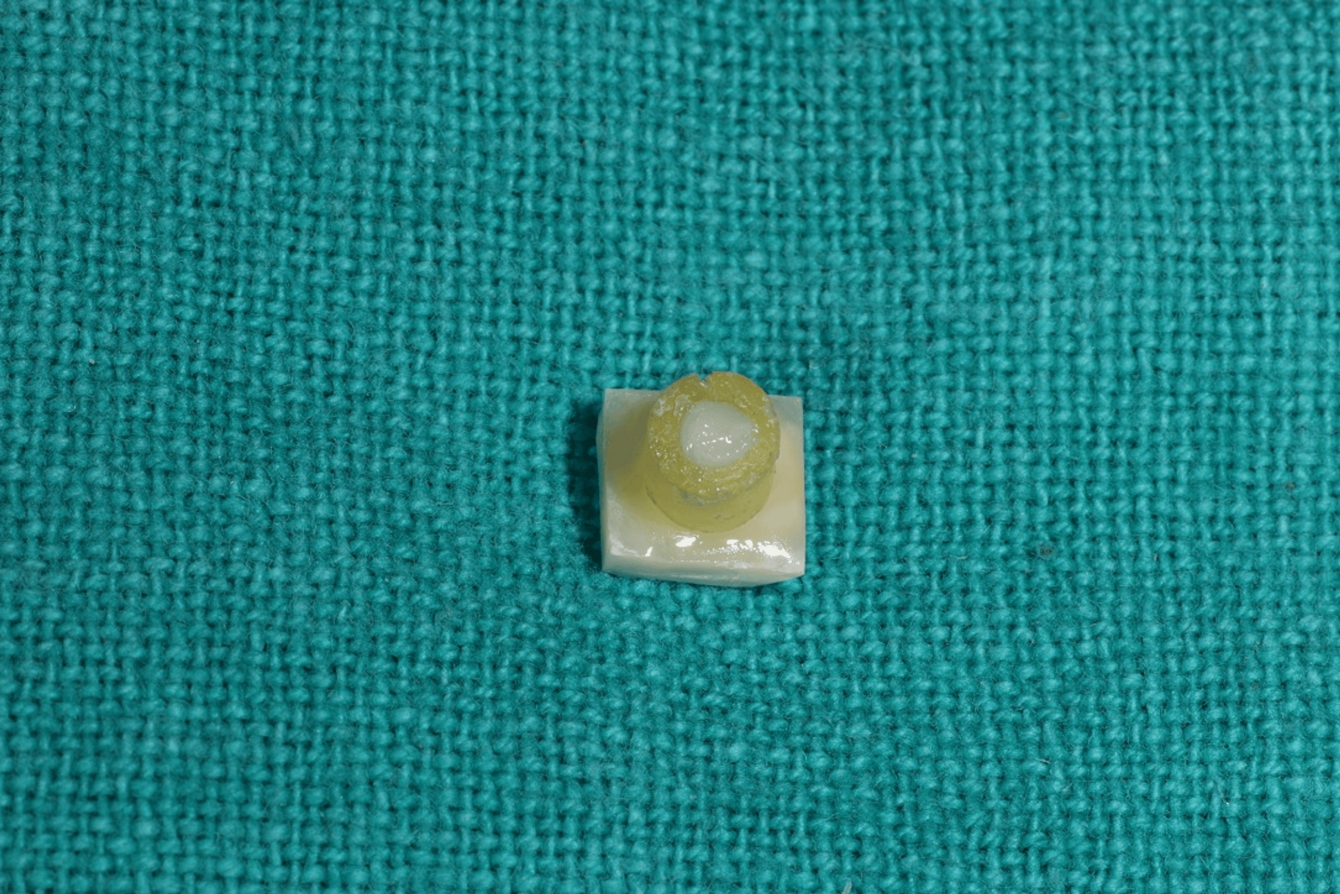
Sample with composite resin

The surface of dentine was gently dried with an air stream, followed by drying using absorbent paper, after which the application of an etch-and-rinse adhesive system (Ivoclar Vivadent, Switzerland) was performed according to the manufacturer’s instructions, followed by the placement of the composite resin (Kulzer, Germany). All the samples were mounted using cold-cure acrylic (Dental Product of India (DPI)) resin (Figure 3).

In all four groups, a transparent Teflon cylinder of dimensions (2.65 mm diameter × 3 mm height) was positioned on the prepared tooth surface to serve as a mould for composite placement. The composite resin was light-cured for 20 seconds from three directions from the occlusal surface and two from the mesial and distal sides, using an LED curing unit (Woodpecker L15000, China) with the tip positioned approximately 2 mm from the composite surface, following the manufacturer’s instructions. The LED unit emits a narrow wavelength spectrum (430-490 nm), efficiently activating photoinitiators in composites to achieve uniform polymerization. After the curing cycle was completed, the specimens were kept in distilled water at room temperature for 30 days. 

Shear bond strength analysis

The SBS was measured using a Universal Testing Machine (Zwick/Roell Z020, ZwickRoell Group, Germany). The specimens were positioned in a mounting jig, and a chisel with an edge was attached to the cross-head to apply a shearing load at a rate of 0.5 mm/min until the bond failure occurred at the resin-dentin interface. The force applied to the specimen was parallel to the bonded interface and adjacent to the dentin surface, as well as perpendicular to the long axis of the tooth, until failure occurred. The specimens were connected to the load-measuring cell, which continuously recorded the applied load throughout testing.

SBS (σ) was calculated using the parameters like the load at the junction of failure, Force (N), and the adhesive area A (mm2): σ in (MPa) = F/A. The results obtained were documented in a Microsoft Excel (Microsoft Corporation, Redmond, Washington, United States), analyzed, and then subjected to statistical analysis. 

Statistical analysis

**Figure 4:**
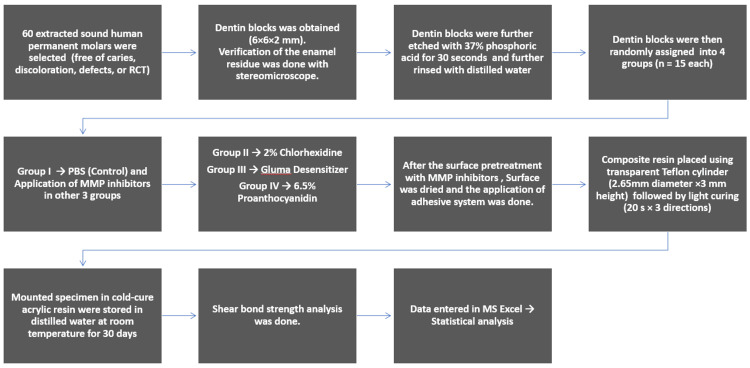
Study flow RCT: root canal treatment; PBS: phosphate-buffered saline; MMP: matrix metalloproteinase

Data were analyzed using software IBM SPSS Statistics for Windows, Version 26 (Released 2018; IBM Corp., Armonk, New York, United States). Means and standard deviations were determined for each group. A Kruskal-Wallis test and post-hoc Mann-Whitney U tests with Bonferroni correction were subsequently employed to identify specific intergroup differences. For all statistical analyses, p < 0.05 was considered statistically significant. The study flow diagram summarizes the process (Figure 4).

## Results

The descriptive analysis of SBS of composites to permanent tooth dentin was expressed in megapascals (MPa), under various conditions where dentin was pretreated. The effect of different dentin pretreatment agents on the SBS of composite resin to permanent tooth dentin was expressed in Newton (N) and MPa.

**Figure 5:**
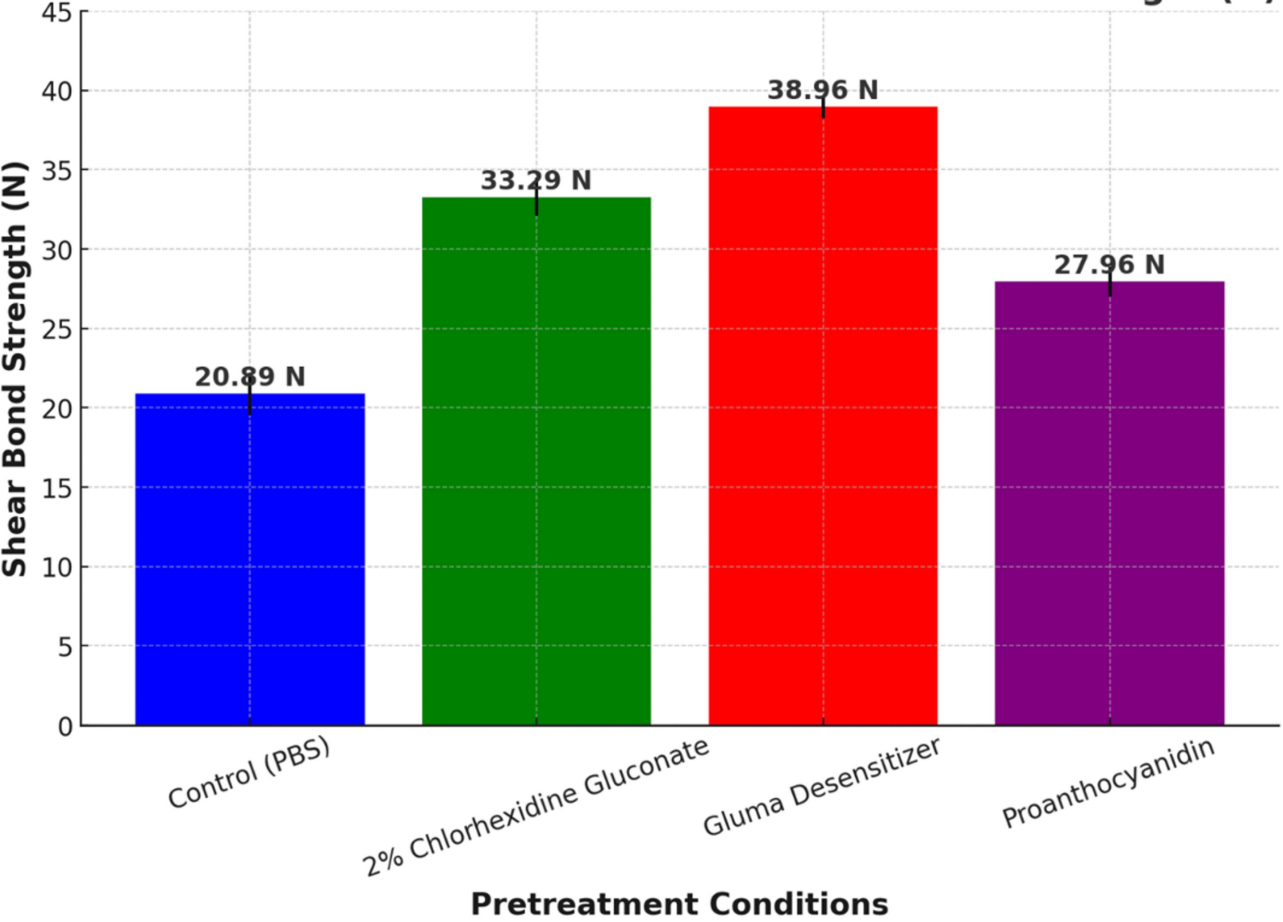
Graphical representation of different pre-treatment conditions in Newtons (N)

Figure 5 represents the mean SBS (N) of composite resin to dentin under different pretreatment conditions, with error bars showing standard deviation. Gluma desensitizer (38.96 N) had the highest bond strength, confirming its superior adhesion-enhancing properties. Around 2% CHX gluconate (33.29 N) significantly improved bond strength, though it was slightly lower than Gluma. Proanthocyanidin (27.96 N) provided moderate improvement over the control but was less effective than the other agents. PBS (20.89 N, control group) had the lowest bond strength, indicating minimal adhesion enhancement. This data confirmed that Gluma desensitizer was the most effective pretreatment agent, followed by 2% CHX gluconate and proanthocyanidin, with the control group showing the weakest bonding performance.

The study evaluated that the control group (PBS) exhibited the lowest mean SBS (6.96 MPa), indicating minimal enhancement of bonding, and had the highest variability (std. dev. = 0.44 MPa) among all groups, suggesting inconsistent bonding performance. In addition to SD, the coefficient of variation (CV) was calculated for all groups to represent relative variability more accurately. The CV values (control = 6.4%, CHX 3.3%, Gluma = 1.8%, proanthocyanidin = 3.2%) demonstrate that Gluma desensitizer had the most consistent results, while the control group exhibited the greatest variability.

The descriptive analysis of SBS demonstrated that Gluma desensitizer achieved the highest mean bond strength (12.98 ± 0.24 MPa). Among all groups, Gluma desensitizer displayed the least variability (SD = 0.24 MPa), confirming its consistency and reliability, whereas the control group showed the highest variability (SD = 0.45 MPa). Control (6.96 ± 0.45 MPa; 95% CI: 6.70-7.22), CHX (11.12 ± 0.37 MPa; 95% CI: 10.91-11.33), Gluma (12.98 ± 0.24 MPa; 95% 12.84-13.12), and proanthocyanidin (9.32 ± 0.30 MPa; 95% CI: 9.15-9.49). The descriptive analysis of SBS demonstrated that Gluma desensitizer achieved the highest mean bond strength (12.98 ± 0.24 MPa), followed by 2% CHX gluconate (11.12 ± 0.37 MPa), proanthocyanidin (9.32 ± 0.30 MPa), and the control group (PBS), which recorded the lowest mean SBS (6.96 ± 0.45 MPa). Among all groups, Gluma desensitizer displayed the least variability (SD = 0.24 MPa), confirming its consistency and reliability, whereas the control group showed the highest variability (SD = 0.45 MPa). To provide a clearer picture of variability, the CV was calculated: control = 6.4%, CHX = 3.3%, Gluma = 1.8%, and proanthocyanidin = 3.2%. These results reinforce the superior uniformity of Gluma desensitizer in maintaining consistent bond strength outcomes.

**Figure 6:**
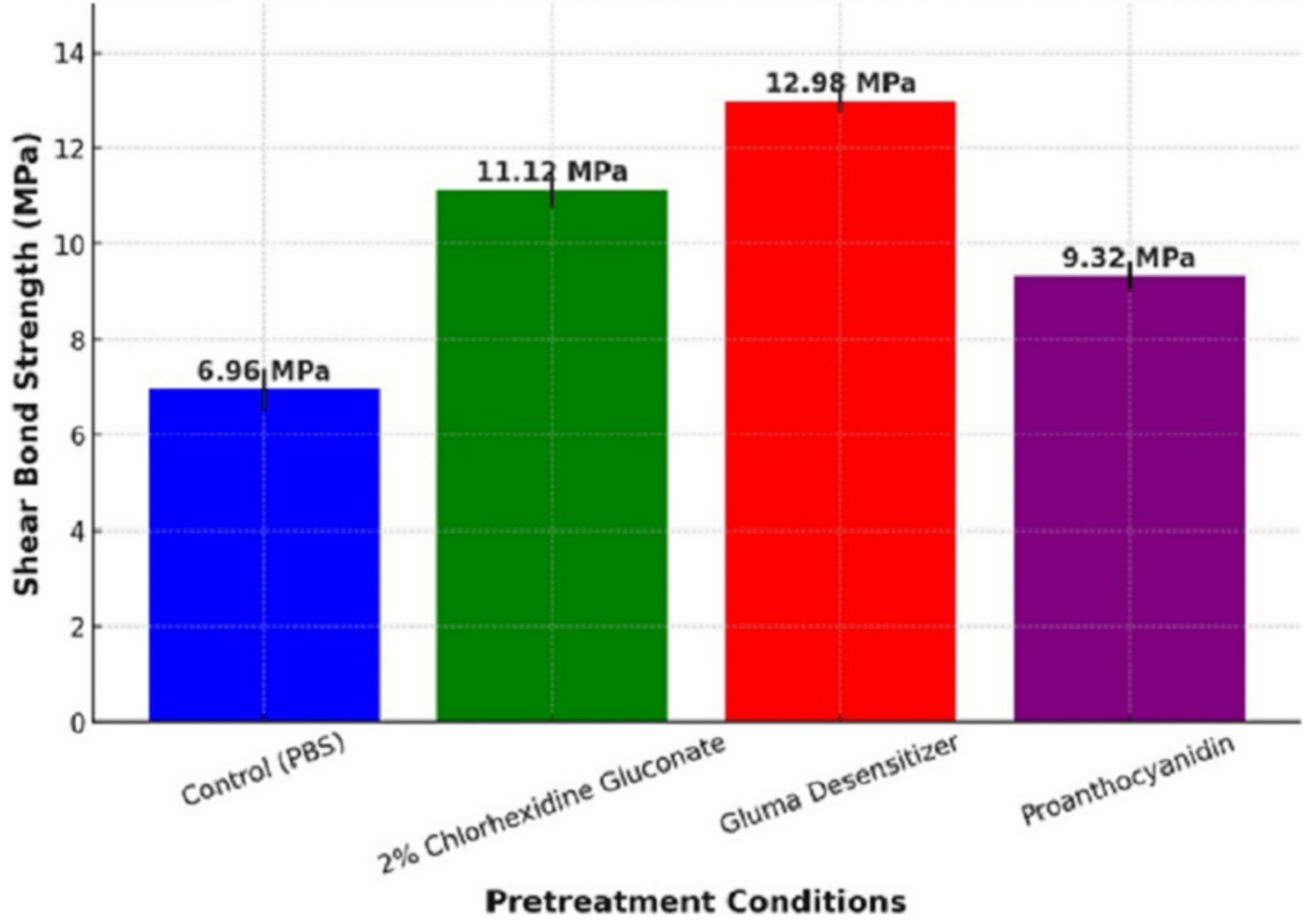
Graphical representation of different pre-treatment conditions in megapascals (MPa)

The data depicted in Figure 6 shows the evaluation of the influence of different agents used in the study, namely 2% CHX gluconate, Gluma desensitizer, and 6.5% proanthocyanidin, and their effect on the SBS of composite resins when bonded to dentin of the permanent tooth, expressed in MPa. Figure 6 represents the mean SBS (MPa) of composite resin to dentin under different pretreatment conditions, with error bars representing standard deviation. Gluma desensitizer (12.98 MPa) provided the highest bond strength, with the least variability, making it the most reliable bonding agent. Around 2% CHX gluconate (11.12 MPa) also significantly enhanced bond strength compared to the control group. Proanthocyanidin (9.32 MPa) offered moderate bond strength improvement but was less effective than Gluma and CHX. PBS (6.96 MPa, control Group) resulted in the weakest bond strength, emphasizing the need for pretreatment.

These results highlight that Gluma desensitizer leads to the strongest and most consistent bond strength, followed by 2% CHX gluconate, while proanthocyanidin has a moderate effect. The control group exhibited the weakest adhesion, reinforcing the importance of dentin pretreatment for optimal bonding performance.

Pairwise comparisons demonstrated statistically significant differences among the evaluated dentin pretreatment agents (p-value <0.05). Table 1 shows the statistical analysis.

**Table 1:** Representing the statistical analysis * indicates statistical significance of observed values; a: Lilliefors significance correction

Tests of Normality						
	Kolmogorov-Smirnov^a^			Shapiro-Wilk		
	Statistic	df	Sig.	Statistic	df	Sig.
Effect of phosphate-buffered saline (control group) on the shear bond strength of the composite attached to permanent teeth dentin, measured in units (N)	.279	15	.003*	.839	15	.012*
Effect of phosphate-buffered saline (control group) on the shear bond strength of the composite attached to permanent teeth dentin, measured in units (Mpa)	.280	15	.002*	.838	15	.012*
Effect of 2% chlorhexidine gluconate on the shear bond strength of the composite attached to permanent teeth dentin, measured in units (N)	.280	15	.002*	.765	15	.001*
Effect of 2% chlorhexidine gluconate on the shear bond strength of the composite attached to permanent teeth dentin, measured in units (Mpa)	.277	15	.003*	.796	15	.003*
Effect of Gluma desensitizer on the shear bond strength of the composite attached to permanent teeth dentin, measured in units (N)	.230	15	.032*	.850	15	.018*
Effect of Gluma desensitizer on the shear bond strength of the composite attached to permanent teeth dentin, measured in units (Mpa)	.229	15	.033*	.849	15	.017*
Effect of proanthocyanidin on the shear bond strength of the composite attached to permanent teeth dentin, measured in units (N)	.267	15	.005*	.857	15	.022*
Effect of proanthocyanidin on the shear bond strength of the composite attached to permanent teeth dentin, measured in units (Mpa)	.265	15	.006*	.855	15	.021*

Overall, these findings suggested that Gluma desensitizer is more effective in enhancing the SBS of composite resins when bonded to tooth dentin when compared to 2% CHX gluconate and 6.5% proanthocyanidin. Based on the above results obtained, the null hypothesis was rejected, as significant differences were observed in SBS among all tested groups. Significant intergroup differences were observed: Gluma and CHX, proanthocyanidin, and control (p = 0.000); CHX and proanthocyanidin and control (p = 0.000); and proanthocyanidin and control (p = 0.000). These results clarify which pairs differed significantly.

Reliability was assessed using a two-way mixed-effects intraclass correlation coefficient (ICC), absolute agreement, and single-measure ICC (3,1), calculating mean difference (bias) and 95% limits of agreement (LoA). An ICC ≥ 0.75 was considered good, while ≥ 0.90 was interpreted as excellent reliability. Intra-examiner agreement for SBS measurements was excellent, with ICC (3,1) = 0.964 (95% CI: 0.905-0.989). Bland-Altman analysis showed a mean bias of +0.03 MPa, with 95% limits indicating minimal systematic error.

A Kruskal-Wallis test indicated a highly significant difference among the four experimental groups (p = 0.000). Post-hoc Mann-Whitney U tests with Bonferroni correction were subsequently employed to identify specific intergroup differences (Table 2).

**Table 2:** Comparative analysis of shear bond strength measurements evaluating the impact of unit selection (Newton) across various dentin pre-treatments *represents statistically significant outcomes

	Pairwise Comparison	N	Mean Rank	Sum of Ranks	P-value
Comparison between N unit and Mpa unit for phosphate-buffered saline (control group) on the shear bond strength of the composite attached to the permanent teeth dentin measure	Results obtained for phosphate-buffered saline (control group) in N unit	15	23.00	345.00	.000*
	Results obtained for phosphate-buffered saline (control group) in Mpa unit	15	8.00	120.00	
	Total	30			
Comparison between N unit and Mpa unit for 2% chlorhexidine gluconate on the shear bond strength of the composite attached to the permanent teeth dentin measure	Results obtained for 2% chlorhexidine gluconate group in N unit	15	23.00	345.00	.000*
	Results obtained for 2% chlorhexidine gluconate in Mpa unit	15	8.00	120.00	
	Total	30			
Comparison between N unit and Mpa unit for Gluma desensitizer on the shear bond strength of the composite attached to the permanent teeth dentin measure	Results obtained for Gluma desensitizer in N unit	15	23.00	345.00	.000*
	Results obtained for Gluma desensitizer in Mpa unit	15	8.00	120.00	
	Total	30			
Comparison between N unit and Mpa unit for proanthocyanidin on the shear bond strength of the composite attached to the permanent teeth dentin measure	Results obtained for proanthocyanidin in N unit	15	23.00	345.00	.000*
	Results obtained for proanthocyanidin in Mpa unit	15	8.00	120.00	
	Total	30			

The results demonstrated that Gluma desensitizer exhibited significantly higher SBS values than 2% CHX gluconate, proanthocyanidin, and the control group (p = 0.000). (Table 3)

**Table 3:** Comparative analysis of shear bond strength measurements evaluating the impact of unit selection (megapascal) across various dentin pre-treatments *represents statistically significant outcomes

	Pairwise Comparison	Mean	Std. Deviation	P-value
Effect of phosphate-buffered saline (control group) on the shear bond strength in units (Mpa)	Effect of 2% chlorhexidine gluconate on the shear bond strength in unit (Mpa)	11.1160	.37236	.000*
	Effect of Gluma desensitizer on the shear bond strength in unit (Mpa)	12.9847	.23712	.000*
Effect of 2% chlorhexidine gluconate on the shear bond strength in units (Mpa)	Effect of Gluma desensitizer on the shear bond strength in unit (Mpa)	12.9847	.23712	.000*
	Effect of proanthocyanidin on the shear bond strength in unit (Mpa)	9.3187	.30199	.000*
Effect of Gluma desensitizer on the shear bond strength in units (Mpa)	Effect of proanthocyanidin on the shear bond strength in unit (Mpa)	9.3187	.30199	.000*
	Effect of 2% chlorhexidine gluconate on the shear bond strength in unit (Mpa)	11.1160	.37236	.000*

Around 2% CHX gluconate also performed better than proanthocyanidin and the control (p = 0.000). Proanthocyanidin showed a statistically significant improvement in SBS compared to the control group (p = 0.000). 

The Kolmogorov-Smirnov and Shapiro-Wilk tests showed significant p-values (< 0.05) for all treatment groups, indicating that the SBS data did not follow a normal distribution. Consequently, non-parametric statistical tests were applied for inferential analysis. These findings collectively confirm that all pretreatments improved bonding performance compared to untreated dentin, with Gluma desensitizer producing the strongest and most consistent adhesive interface. These results clarify which pairs differed significantly.

Reliability and calibration were emphasized to ensure that the measurements reflected true consistency rather than operator- or equipment-related variability. Standardization and examiner calibration before specimen testing reduce subjective bias and enhance the reproducibility of results. By repeating measurements under controlled conditions, using ICC and Bland-Altman analysis, we confirmed that intra-examiner variability was negligible. This strengthens the credibility of the findings, as high reliability (ICC = 0.964) and minimal bias (+0.03 MPa) demonstrate that the observed differences in SBS are attributable to material behavior rather than measurement error.

## Discussion

Adhesion to dentin presents a more challenging and demanding task compared to the process of bonding with enamel, and the challenge is further elevated if there is the presence of partially encapsulated type I collagen meshwork in the interfacial hybrid zone [16]. The acid etching process in dentin exposes enzymes like matrix-bound proteases, namely cysteine cathepsins and MMPs, leading to hydrolytic and enzymatic degradation of the collagen, which ultimately leads to compromised bonding strength at the bonding interface [17,18]. MMP inhibitors are therefore more strategically and carefully utilized as dentin pretreatments to effectively impede activation of MMP and increase the SBS, which results in increasing the longevity of the restoration [15,18]. 

In the present in vitro study, it was observed that the MMP inhibitors increased the SBS of dentin significantly. While Gluma desensitizer produced superior efficacy in increasing the SBS of composites to dentin using the etch and rinse adhesive system when compared to the control group, 2% CHX, and 6.5% proanthocyanidin, thereby rejecting the null hypothesis. The mean SBS value range of 12.98 MPa for Gluma desensitizer in this study was in complete accordance with the findings from other research investigating the SBS of Gluma when applied to permanent teeth dentin. While 2% CHX gluconate demonstrated a reduced bond strength in comparison to Gluma desensitizer, it still performed better than 6.5% proanthocyanidin, showing a significant difference in the values [18].

In the field of dentistry, both Gluma and CHX [18,19] serve as MMP inhibitors to improve the stability and strength of resin-dentin bonds. Gluma, a glutaraldehyde-based desensitizer, comprises 5% glutaraldehyde and 35% HEMA and inhibits MMPs through crosslinking [20]. Whereas CHX acts as a non-specific protease inhibitor of both the enzymes, like MMPs and cysteine cathepsins. CHX is thought to bind to various proteins via the cation chelation mechanism, thereby inhibiting the catalytic activity of MMPs by binding with zinc or calcium ions [15]. Glutaraldehyde in Gluma, on the other hand, reacts with plasma proteins like albumin, which causes precipitation on the dentinal surface. These precipitates interact with HEMA to form a copolymer named poly-HEMA, which is a cross-linked product of glutaraldehyde with albumin, leading to dentinal tubule blockage [20,21]. Despite this surface deposition, HEMA is found to accelerate the process of adhesive monomer penetration into dentin [21].

Glutaraldehyde is also known to act as a cross-linking agent, thereby increasing the resistance of un-cross-linked or weakly cross-linked collagen matrices to enzymatic degradation by reacting with its aldehyde groups with amine groups in lysine and hydroxylysine within collagen fibrils in the dentinal tissue. By enhancing dentin's mechanical properties, glutaraldehyde present in Gluma desensitizer helps to preserve the integrity of the hybrid layer, thereby reducing the bond degradation and increasing the bond strength by minimizing the contraction gap formation [20]. While both Gluma and CHX can act as effective MMP inhibitors, it has been noted that usage of CHX with self-etch adhesives is often associated with reduced bond strength, likely because it hinders the adhesive's ability to penetrate the dentin adequately [22]. The type of solution used can also be a factor contributing to the bond strength of self-etch adhesives.

Water-based CHX demonstrates greater effectiveness compared to its ethanol-based formulation, though its efficacy remains lower than that of Gluma. Gluma is considered advantageous in etch-and-rinse systems due to its single-step ability to induce collagen crosslinking, inhibit MMPs, provide dentin desensitization, and enhance bond strength, thereby offering a versatile and potentially superior approach for achieving a stronger and more durable hybrid layer [20]. In our study, 6.5% proanthocyanidin provided a significantly moderate improvement of SBS when compared to the control group, but it was found to be less when compared to 2% CHX. Increased bond strength likely results from a greater number of collagen cross-linkages, enhancing collagen stability. Proanthocyanidins (PAs) are unique due to their four monomer molecules (catechin, ent-catechin, epicatechin, and ent-epicatechin) and various interflavonoid bonds. PAs bind to proline-rich proteins like collagen and enhance proline hydroxylase activity, essential for collagen biosynthesis [23]. Various studies also stated that PA enhances collagen production and stability, strengthens dentin's resistance to enzymatic breakdown, and improves bonding strength and durability [24].

However, studies have shown that varying concentrations of PA (1%, 2.5%, 5%, and 10%) can alter monomer conversion and the polymerization kinetics of Bisphenol A-glycidyl methacrylate (bis-GMA)/HEMA model adhesive, although these changes remain within acceptable limits [25], which is unlikely in 2% CHX. While grape seed extract may stain dentin brown, the long-term durability of bond strength requires further experimental research. Even an in-depth, comprehensive investigation needs to be carried out to explore the effects of cross-linking agents on dentinal MMPs [25].

We acknowledge that thermocycling or other artificial aging protocols (e.g., pH cycling, mechanical loading) may more closely simulate intraoral conditions and could provide additional clinical relevance [26]. However, in this study, 30-day water storage was selected as an approach to monitor baseline SBS without introducing additional variables. 

Limitations of our study included 60 extracted permanent molars, which may have variability across the population. The bonding performance could be influenced by age, structural composition, and factors exposed to the oral environment of the collected patients. In-vitro evaluation does not simulate the oral environment with saliva, masticatory forces, pH, and thermal variations. Storage parameters can influence the outcomes. We have used only one concentration of agents tested in our study. Variations in their concentrations can have different results. Long-term effects of bonding and its clinical implications of postoperative sensitivity and longevity of restoration were not included. 

Notably, future in vivo research should include long-term clinical trials with larger sample sizes that can assess the performance of these MMP inhibitors. This is crucial for developing comprehensive guidelines, which in turn can improve dental adhesive procedures, thereby benefiting long-term patient outcomes in restorative dentistry. Integrated innovative approaches will be essential in overcoming current limitations in the dentin bonding systems. The findings from this study may serve as baseline data for future research.

## Conclusions

Within the limitations of the present study, the application of MMP inhibitors is effective in enhancing the SBS of dentin to composite restorations. Although the observed enhancement in SBS is likely related to MMP inhibition, definitive confirmation of causality would require further validation through enzymatic activity assays. Among the evaluated agents, Gluma desensitizer, which contains glutaraldehyde and HEMA, demonstrated the greatest efficacy. Although proanthocyanidin showed promise as a biocompatible, natural biomodifier, its overall effectiveness remained lower than that of Gluma and CHX. All these findings highlight the clinical relevance of incorporating MMP inhibitors as adjuncts in adhesive dentistry protocols to enhance the longevity and stability of bonded restorations.
